# Distance to Care, Facility Delivery and Early Neonatal Mortality in Malawi and Zambia

**DOI:** 10.1371/journal.pone.0052110

**Published:** 2012-12-27

**Authors:** Terhi J. Lohela, Oona M. R. Campbell, Sabine Gabrysch

**Affiliations:** 1 Department of Anaesthesiology and Intensive Care Medicine, Jorvi Hospital, Helsinki University Hospital, Espoo, Finland; 2 London School of Hygiene & Tropical Medicine, Faculty of Epidemiology and Population Health, London, United Kingdom; 3 University of Heidelberg, Institute of Public Health, Heidelberg, Germany; CUNY, United States of America

## Abstract

**Background:**

Globally, approximately 3 million babies die annually within their first month. Access to adequate care at birth is needed to reduce newborn as well as maternal deaths. We explore the influence of distance to delivery care and of level of care on early neonatal mortality in rural Zambia and Malawi, the influence of distance (and level of care) on facility delivery, and the influence of facility delivery on early neonatal mortality.

**Methods and Findings:**

National Health Facility Censuses were used to classify the level of obstetric care for 1131 Zambian and 446 Malawian delivery facilities. Straight-line distances to facilities were calculated for 3771 newborns in the 2007 Zambia DHS and 8842 newborns in the 2004 Malawi DHS. There was no association between distance to care and early neonatal mortality in Malawi (OR 0.97, 95%CI 0.58–1.60), while in Zambia, further distance (per 10 km) was associated with lower mortality (OR 0.55, 95%CI 0.35–0.87). The level of care provided in the closest facility showed no association with early neonatal mortality in either Malawi (OR 1.02, 95%CI 0.90–1.16) or Zambia (OR 1.02, 95%CI 0.82–1.26). In both countries, distance to care was strongly associated with facility use for delivery (Malawi: OR 0.35 per 10km, 95%CI 0.26–0.46). All results are adjusted for available confounders. Early neonatal mortality did not differ by frequency of facility delivery in the community.

**Conclusions:**

While better geographic access and higher level of care were associated with more frequent facility delivery, there was no association with lower early neonatal mortality. This could be due to low quality of care for newborns at health facilities, but differential underreporting of early neonatal deaths in the DHS is an alternative explanation. Improved data sources are needed to monitor progress in the provision of obstetric and newborn care and its impact on mortality.

## Introduction

While efforts to reduce child mortality have been successful, neonatal mortality remains high, particularly in sub-Saharan Africa [1]. Globally, over 40% of all deaths among children under five are estimated to occur during the first four weeks of life [2], which means that reducing neonatal mortality is crucial for reaching Millennium Development Goal 4. The interventions needed to reduce the approximately 3 million neonatal deaths globally [3], and in particular the estimated 2.2 million early neonatal deaths (i.e. in the first week of life) [4] are “intimately linked to maternal health” and to providing adequate care at birth [5].

In many low-income countries, distances to health facilities can be considerable, particularly in rural areas, and vehicles for transport are rarely available. There have been several studies on the influence of distance from care on child survival [6]–[8], but “the impact of spatial dimensions on neonatal survival has not been thoroughly investigated, even though access to good quality delivery care is considered to be one of the main priorities when trying to reduce neonatal mortality” [9].

In countries with high neonatal mortality, roughly half of all births occur without skilled care and about a third of early neonatal mortality is intrapartum-related [5], [10]. Distance to delivery care and the level of care provided are important determinants of facility delivery, as we recently demonstrated for Zambia [11]. Delivery in a health facility with a skilled provider should reduce early neonatal mortality, as has been shown in some contexts [12], [13]. Facilitating skilled attendance at delivery is thus likely to be a major pathway via which proximity to care can improve early neonatal survival [9], albeit not the only one, as access to care for complications occurring after birth, e.g. neonatal sepsis, is also important.

However, it can be difficult to demonstrate the beneficial impact of facility delivery on early neonatal survival due to confounding by complications during pregnancy or childbirth. In contexts where most deliveries occur at home, those seeking care at facilities may well be complicated cases, with a higher risk of early neonatal death. In Bangladesh, maternal and early neonatal mortality rates were much higher among women delivering in a health facility, especially in a higher-level facility, than among those delivering at home [14]. This difference decreased as the percentage seeking skilled delivery care increased over time [14] – which is consistent with facility deliveries comprising more complicated cases in settings with low care-seeking. Alternatively, it is possible that care in facilities does not improve survival or even that certain practices at health facilities increase mortality. Unfortunately, it is difficult to get valid and comparable data on complications for home and facility deliveries in order to adjust for this. Even in high-income settings, it is impossible to capture all risk factors for complications that direct women towards choosing a facility delivery [15].

One of the reasons why the effects of distance to delivery care and quality of care on early neonatal mortality have rarely been studied is a lack of adequate data. Our approach was to link household data from Demographic and Health Surveys with facility data from Health Facility Censuses in Malawi and Zambia [11]. The aim of this study was (1) to investigate the influence of distance to delivery care and of level of care on early neonatal mortality, (2) to study the influence of distance and level of care on facility delivery, and (3) to explore the influence of facility delivery on early neonatal mortality in the presence of confounding by complications during pregnancy or childbirth.

## Methods

This study was granted ethical approval by the London School of Hygiene & Tropical Medicine ethics committee (application number 5172).

### Datasets

We analysed data from two Demographic and Health Surveys (DHS): the 2004 Malawi DHS and the 2007 Zambia DHS. Information on individual and household characteristics, birth histories, survival of children and place of birth for children born in the five years prior to the survey was collected by interviewing a nationally representative sample of women aged 15–49 years, using two-stage cluster sampling. For children who died, age at death was recorded in days if they were less than one month old. Early neonatal deaths are deaths at age 0–6 days among live-born children [16]. We only included rural births in the analysis (as classified in the DHS), since distance is likely to be more important in rural areas where distances to health facilities are longer and the transport network is weaker than in urban areas.

DHS datasets also contain information on duration of pregnancy for the most recent pregnancy that did not result in a live birth, thus allowing us to identify stillbirths as those with at least seven completed months of pregnancy and to calculate perinatal mortality [17]. Since place of delivery and other relevant variables are not available for stillbirths, we did not use perinatal mortality as our main outcome. However, we performed a sensitivity analysis using perinatal mortality as an outcome.

Facility-level data were obtained from national Health Facility Censuses (HFC) conducted in Malawi in 2002 and in Zambia in 2005. The HFC, developed by the Japan International Cooperation Agency (JICA), is a national-level assessment of the functionality of health system assets [18]. There is no sampling; instead, information is collected on all public and semi-public facilities, as well as major private facilities. Data include the precise location (using GPS), availability, and condition of physical infrastructure and equipment, availability of services, and head counts of health workers.

### Level of Care Classification

We defined two main levels of emergency obstetric care (EmOC) aiming to represent referral-level care, typically provided in hospitals, and first-level care corresponding to care in health centres. Due to differences in the information collected from Malawi and Zambia, our definitions of the two levels of care in these countries also differed.

The 1131 Zambian delivery facilities were grouped into basic and comprehensive EmOC facilities according to their reported capacity to perform eight EmOC signal functions: injectable antibiotics, injectable oxytocics, injectable anticonvulsants, manual removal of placenta, removal of retained products, assisted vaginal delivery, caesarean section and blood transfusion. The level of EmOC was defined as basic in facilities performing the first six functions and as comprehensive in facilities performing all eight functions. In addition, information on opening hours, staffing, electricity availability, and referral capacity was added to the classification. This has been described in detail previously [19].

The 2002 Malawi HFC did not collect information on the signal functions. Therefore, the 446 delivery facilities were classified based on staffing, opening hours, availability of safe blood transfusion services (as per WHO definition [20]) and an operating theatre. Delivery facilities with adequate staffing and opening hours 24-hours per day were considered first-level facilities. Facilities with medical doctors, an operating theatre and a safe blood transfusion service in addition to 24-hour functionality were considered back-up facilities. The classification of facilities in Malawi is shown in Table 1.

**Table 1:** Distribution of services in delivery facilities in Malawi in 2002.

	Facilities offering service	Back-up facilities		First-level facilities	
	(n = 446)	Full	Reduced	Full	Reduced
**Utilities**					
Blood transfusion^a^	10%	X			
Main theatre	12%	X	X		
**Health workers**					
3+ doctors^b^	10%	X			
1+ doctor^b^	20%		X		
3+ skilled attendants^c^	27%	X	X	X	
3+ health workers^d^	41%				X
24 hour presence	66%	X	X	X	
24 hour on-call	90%				X
1+ skilled attendant^c^	92%				X
**Facilities qualifying** e		**32**	**16**	**58**	**72**

a Blood transfusion defined as the availability of blood transfusion service and the ability to test blood for hepatitis B, HIV and syphilis^1^.

b Includes doctors and clinical officers.

c Includes skilled delivery attendants defined as doctors, clinical officers, midwives or midwife/nurses.

d Includes doctors, clinical officers, midwives, midwife/nurses, medical assistants, nurses and matrons.

e There were a total of 446 facilities offering delivery care. The remaining 268 facilities (60%) did not fulfill even reduced first-level criteria.

### Distance Calculation

We measured straight-line distances from the Malawi 2004 and the Zambia 2007 rural DHS clusters to the closest health facilities of various levels. Distance measurement was done in the GIS platform ArcView 3.2 (ESRI) with the “Nearest Neighbor 3.6″ extension, using the geographic coordinates of DHS clusters and health facilities. Clusters without geographic data were excluded from the analysis. As geographic coordinates were available on the current place of residence, births that occurred before the motheŕs moving to the current location were also excluded from the analysis. This was the case for 700 out of 9542 (7%) Malawian and 466 out of 4237 (11%) Zambian births. In a process called “geo-scrambling”, Macro International misplaces the coordinates of DHS clusters to protect the confidentiality of the cluster individuals, which introduces an error of up to 5 km to the distance measurements [21]. Therefore, and because we lacked data on roads and terrain, a precise estimation of travel time could not be made. We used distance as a linear effect (per 10 km) in order to have comparable models for the two countries, although for Zambia, where distances are long, a logarithmic transformation of distance would have been more appropriate.

### Conceptual Framework

**Figure 1:**
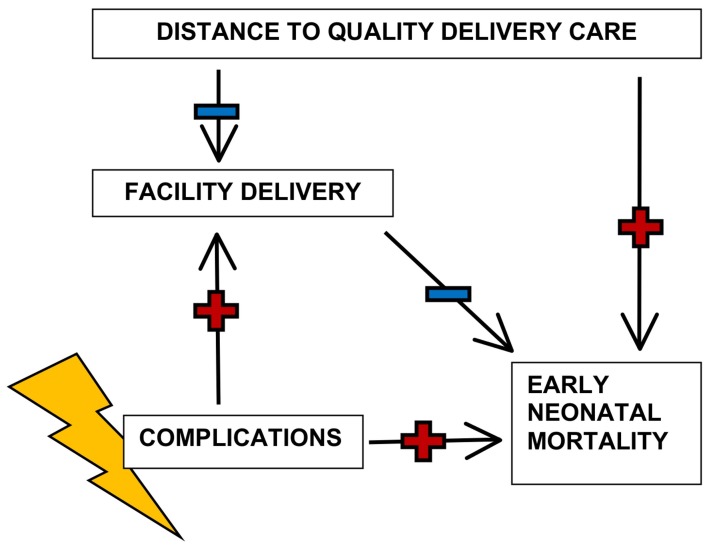
Conceptual framework of the relationships between distance, facility delivery, early neonatal mortality and delivery complications. Distance to care can impact neonatal mortality either by influencing place of delivery, or directly, i.e. via other, unspecified pathways (e.g. care-seeking for neonatal sepsis). Complications during pregnancy or childbirth (which are hard to measure) influence both place of delivery and neonatal mortality, and thus confound the association between facility delivery and neonatal mortality.

The conceptual framework presented in Figure 1 guided this analysis. Our primary outcome was early neonatal mortality, defined as a death within the first seven days of life. The main exposures were distance to delivery care and the level of care provided at the facilities. We also studied the effects of distance and level of care on facility delivery and the effect of facility delivery on early neonatal mortality, to explore the role of facility delivery as a mediating factor between distance to delivery care and early neonatal mortality.

As illustrated by the conceptual framework (Figure 1), the association between facility delivery and early neonatal mortality is likely to be confounded by complications during pregnancy or childbirth. In settings where “uptake of skilled birth attendance […] is low, women will only seek care when they are ill, and they may do so too late” [14], also evident from the fact that in low-income countries, near-miss cases often arrive at a health facility already in a critical condition [14], [22], [23]. Thus, where facility delivery is rare, the proportion of obstetric emergencies among facility deliveries is likely to be higher than in communities where delivering in a health facility is common and facilities are also used for normal deliveries. In settings where the majority of facility deliveries are complicated cases, the odds of early neonatal death may even be higher among facility births than among home births because of this adverse selection into facilities [14]. Without valid data on complications, it is not meaningful to study the association between facility delivery and early neonatal mortality on an individual level.

To get around this problem, we stratified by frequency of facility delivery in the sampling cluster (usually a village in rural areas) as a proxy for the proportion of obstetric emergencies among facility deliveries. We created four strata, aiming to have the bottom and top 15% separate, while ensuring that no stratum contained less than 5% of newborns. This required a cut-off at 70% in Zambia. In this context of the cluster-level analysis, we also investigated the proportion of caesarean sections and hospital deliveries among facility deliveries as indicators of complications, comparing clusters with differing frequencies of facility delivery.

### Statistical Analysis

The samples for the mortality analysis comprised 8842 newborns in Malawi and 3771 newborns in Zambia. For the facility delivery analysis, we included only the firstborn for multiple births, leaving 8537 deliveries in Malawi, and 3682 deliveries in Zambia. A large number of variables on the individual, household and cluster level were considered as potential confounders of the associations between distance and early neonatal mortality, and between distance and facility delivery (see Table S1). Variables that could be on the causal pathway (e.g. antenatal care use) or that may be affected by reverse causality (e.g. breastfeeding of the baby) were not considered as potential confounders.

Variables that changed the logOR of the associations of interest by at least 10% were considered confounders. These variables were then included one by one in descending order of magnitude in a multivariable regression model using robust standard errors to take account of clustering. A variable was kept in the model if adding it changed the logOR of distance or level of care by at least 10%. Altogether four multivariable regression models were built using this forward fitting procedure, for both outcomes – early neonatal mortality and facility delivery – and both countries – Malawi and Zambia.

## Results

Of 8842 rural live births in the Malawian sample, 198 died during the first week of life (22 per 1000). In the Zambian sample of 3771 rural live births, 96 early neonatal deaths occurred (26 per 1000). Half of the Malawian and one third of the Zambian newborns were delivered in a health facility. In Zambia, 14% of the sample population lived more than 15 km from a delivery facility, whereas in Malawi the comparable figure was less than 2%. (Table 2).

**Table 2:** Crude associations between (A) early neonatal mortality and (B) facility delivery and distance to delivery services and level of care.

			A		B	
	Newborns(%)	Early neonatal deaths	Early neonatal mortality (per 1000)	Crude OR (95%CI),p-value^a^	Facility delivery^b^ (%)	Crude OR (95%CI),p-value^a^
**Distance to delivery services**						
**Malawi**	**n = 8842**	**n = 198**	**22**	**p = 0.40**	**52.1**	**p<0.001**
**0–2 km**	856 (9.7)	18	21	0.99 (0.55–1.79)	68.0	1.41 (0.96–2.06)
**2–5 km**	3433 (38.8)	73	21	1	60.1	1
**5–10 km**	3262 (36.9)	76	23	1.1 (0.74–1.64)	47.0	0.59 (0.48–0.72)
**10–15 km**	1148 (13.0)	25	22	1.0 (0.63–1.66)	34.0	0.34 (0.26–0.45)
**>15 km**	143 (1.6)	6	42	2.0 (1.17–3.48)	28.3	0.26 (0.17–0.39)
**Zambia**	**n = 3771**	**n = 96**	**26**	**p = 0.04**	**32.5**	**p<0.001**
**0–2 km**	432 (11.5)	15	35	1.0 (0.46–2.39)	39.2	0.88 (0.56–1.40)
**2–5 km**	1021 (27.1)	34	33	1	42.1	1
**5–10 km**	1072 (28.4)	24	22	0.66 (0.35–1.26)	30.4	0.60 (0.42–0.85)
**10–15 km**	716 (19.0)	14	20	0.58 (0.33–1.03)	24.6	0.45 (0.29–0.69)
**>15 km**	530 (14.1)	9	17	0.50 (0.22–1.14)	23.6	0.42 (0.25–0.72)
**Level of care within 15 km**						
**Malawi**	**n = 8842**	**n = 198**	**22**	**p = 0.55**	**52.1**	**p = 0.07**
** None**	143 (1.6)	6	42	2.0 (1.10–3.64)	28.3	0.42 (0.27–0.65)
** Substand.**	1913 (21.6)	41	21	1	48.3	1
** Red. first**	1386 (15.7)	31	22	1.04 (0.59–1.85)	50.7	1.10 (0.79–1.53)
** Full first**	2046 (23.1)	52	25	1.19 (0.74–1.92)	57.1	1.42 (1.10–1.83)
** Red backup**	1127 (12.8)	22	20	0.91 (0.55–1.50)	54.1	1.26 (0.93–1.71)
** Full backup**	2227 (25.2)	46	21	0.96 (0.56–1.67)	52.3	1.17 (0.90–1.53)
**Zambia**	**n = 3771**	**n = 96**	**26**	**p = 0.51**	**32.5**	**p<0.001**
** None**	530 (14.1)	9	17	0.58 (0.24–1.44)	23.6	0.75 (0.43–1.31)
** Substand.**	767 (20.3)	22	29	1	29.2	1
** BEmOC-4**	781 (20.7)	14	18	0.62 (0.25–1.55)	26.4	0.87 (0.57–1.34)
** BEmOC-2**	608 (16.1)	25	41	1.45 (0.77–2.73)	36.5	1.39 (0.88–2.21)
** BEmOC(-1)**	598 (15.9)	15	25	0.87 (0.40–1.90)	36.0	1.36 (0.93–2.00)
** CEmOC (-1)**	487 (12.9)	11	23	0.78 (0.32–1.90)	48.2	2.26 (1.41–3.62)

a P-values are from tests for trend over categories of distance, or categories of level of care.

b n = 8679 in Malawi, n = 3682 in Zambia. Only included the first child of multiple births. Information on delivery place missing for 12 births in Malawi and 10 births in Zambia.

There was no association between distance to care and early neonatal mortality in Malawi, either crudely or adjusted for a wide range of confounders (OR 0.97, p = 0.89), while in Zambia, longer distance (per 10 km) was associated with lower early neonatal mortality both without and with control for confounding (OR 0.55, p = 0.01). The level of care provided in the closest facility showed no crude or adjusted association with early neonatal mortality in either of the two countries (Table 2A, Table 3A). A sensitivity analysis using perinatal mortality instead of early neonatal mortality (including 156 rural stillbirths in Malawi and 48 in Zambia) yielded virtually identical odds ratios in the analysis of the Malawian data and for level of care in Zambia, while the adjusted odds ratio for distance in Zambia was less extreme (OR 0.66, p = 0.04).

**Table 3:** Crude and adjusted associations (ORs and 95% CI) between (A) early neonatal mortality and (B) facility delivery and distance to delivery services and level of care.

	A		B	
	Early neonatal mortality^a^		Facility delivery^a^	
	Malawi^b^	Zambia^c^	Malawi^d^	Zambia^e^
	n = 8260	n = 3019	n = 8416	n = 3682
**Crude model:**				
**Distance to closest delivery facility**(linear effect, per 10 km)	**1.08** (0.70–1.68),p = 0.72	**0.61** (0.39–0.96),p = 0.03	**0.28** (0.21–0.36),p<0.001	**0.67** (0.50–0.89),p = 0.005
**Level of care at closest delivery facility or 5 km there of**(linear effect, per category higher)	**0.99** (0.88–1.12),p = 0.90	**1.04** (0.83–1.29),p = 0.75	**1.05** (0.98–1.12),p = 0.14	**1.19** (1.07–1.32),p = 0.001
**Adjusted for confounders**	**n = 8260**	**n = 3019**	**n = 8416**	**n = 3682**
**Distance to closest delivery facility**(linear effect, per 10 km)	**0.97** (0.58–1.60),p = 0.89	**0.55** (0.35–0.87),p = 0.01	**0.35** (0.26–0.46),p<0.001	**0.73** (0.57–0.94),p = 0.01
**Level of care at closest delivery facility or 5 km there of**(linear effect, per category higher)	**1.02** (0.90–1.16),p = 0.74	**1.02** (0.82–1.26),p = 0.87	**0.99** (0.93–1.05),p = 0.66	**1.12** (1.00–1.24),p = 0.04

a sample sizes are reduced due to missing values of some confounding variables.

b confounding variables: meńs opinion on female autonomy in cluster, ethnicity, partneŕs occupation, partneŕs education, womeńs media use in cluster, education, wanted pregnancy, siblings under 7 years old in household, estimate of newborn size (by mother), meńs media use in cluster, womeńs mobility autonomy in cluster, language, womeńs financial autonomy in cluster, multiple pregnancy, occupation, marital status, age at birth, modern attitudes, meńs modern attitudes in cluster, exposure to health programmes in the media, media use, sex of index child.

c confounding variables: partneŕs education in years, relationship autonomy, partneŕs occupation, media use, womeńs financial autonomy in cluster, wealth, womeńs relationship autonomy in cluster, modern attitudes, newborn size estimate (by mother), marital status, occupation, household composition and siblings under 7 years old, education, literacy, womeńs mobility autonomy in cluster.

d confounding variables: wealth, womeńs relationship autonomy in cluster, partneŕs education in years, education in years, partneŕs occupation, meńs opinion on female autonomy in cluster, womeńs modern attitudes in cluster, womeńs financial autonomy in cluster, womeńs autonomy to seek health care in cluster.

e confounding variables: womeńs relationship autonomy in cluster, meńs modern attitudes in cluster, language, wealth, womeńs autonomy to seek health care in cluster, meńs opinion on female autonomy in cluster.

To understand these unexpected results better, we studied facility use for delivery as a key mediating factor between distance to a facility and early neonatal mortality (Figure 1). We knew from previous work that longer distance to a facility and lower level of care at the closest facility were both associated with lower odds of facility delivery in Zambia [11]. We found that in Malawi, the association between distance to care and facility use was even stronger than in Zambia: the odds of facility delivery decreased by 65% for every 10 km increase in distance to the closest facility (OR 0.35, p<0.001). Unlike in Zambia, level of care at the closest facility was not associated with facility use for delivery in Malawi (Table 2B, Table 3B).

As a second step, we wanted to investigate whether facility delivery (as compared to home delivery) was associated with lower early neonatal mortality. To overcome the problem of confounding by complications, i.e. that health facilities attract complicated births with a higher risk of early neonatal mortality (Figure 1), we stratified by frequency of facility use in the cluster. In settings where less than 15% of women in a cluster deliver in a health facility, those that did were much more likely to give birth in a hospital (as opposed to a health centre) and by caesarean section, both in Zambia and in Malawi (Table 4), indicating that indeed a high proportion of these births are likely to have been seeking emergency care for complications.

**Table 4:** Percentage of deliveries in hospital and by caesarean section among facility deliveries, by frequency of facility delivery in the cluster.

Facility deliveries in cluster	Deliveries (%)	Facility deliveries (%)	Hospital^a^ deliveries among facility deliveries (%)	Delivery by C-section among facility deliveries (%)
**Malawi**			**p = 0.003** b	**p = 0.004** b
** Unstratified**	**8679 (100)**	**4525 (52.1)**	**1823 (40.3)**	**211 (4.7)**
** <15%**	461 (5.3)	40 (8.7)	26 (65.0)	6 (15.0)
** 15–50%**	3578 (41.3)	1212 (33.9)	502 (41.4)	66 (5.5)
** 50–85%**	3697 (42.6)	2416 (65.4)	974 (40.3)	107 (4.4)
** >85%**	943 (10.9)	857 (90.9)	321 (37.5)	32 (3.7)
**Zambia**			**p<0.001** b	**p = 0.02** b
** Unstratified**	**3682 (100)**	**1198 (32.4)**	**225 (21.3)**	**55 (4.6)**
** <15%**	954 (25.9)	69 (7.2)	22 (31.9)	10 (14.5)
** 15–50%**	1867 (50.7)	574 (30.7)	146 (25.4)	23 (4.0)
** 50–70%**	657 (17.9)	387 (58.9)	64 (16.5)	13 (3.4)
** >70%**	204 (5.5)	168 (82.4)	23 (13.7)	9 (5.4)

a In Zambia, hospital = government hospital (mission and private not separate).

b P-values from Chi square test.

Distances to the closest delivery facility were longer for clusters where delivery care-seeking is rare and in Zambia, the level of care available within 15 km was lower for these clusters (Table 5, left). However, early neonatal mortality did not differ significantly by frequency of facility delivery in the cluster (Table 5, column “All”; chi-square p-values 0.86 for Malawi and 0.31 for Zambia): In clusters with low facility delivery, it was 19 per 1000 in Malawi and 20 in Zambia, and in clusters with high facility delivery, it was 22 per 1000 in Malawi and 24 in Zambia.

**Table 5:** Early neonatal mortality, by place of delivery and by frequency of facility delivery in the cluster.

Facility deliveriesin cluster	Newborns insample (%)	Average distance todelivery facility (km)	Average quality of carewithin 15 km (score^a^)	Early neonatal mortality(per 1000) among			OR (95%CI) of facility vshome delivery
				All	Facility births	Home births	
**Malawi**	**n = 8830**						**p-value = 0.29** b
**Unstratified**	**100**	**5.9**	**3.0**	**22**	**21**	**24**	**0.86 (0.65–1.14)**
** <15%**	5.3	8.1	3.3	19	25	19	1.33 (0.16–10.99)
** 15–50%**	41.3	7.2	2.7	21	14	25	0.54 (0.32–0.94)
** 50–85%**	42.6	4.9	3.2	24	24	23	1.04 (0.67–1.62)
** >85%**	10.8	3.6	3.1	22	21	35	0.58 (0.17–2.02)
**Zambia**	**n = 3761**						**p-value = 0.08** b
** Unstratified**	**100**	**8.3**	**2.4**	**25**	**29**	**22**	**1.33 (0.87–2.04)**
** <15%**	25.9	10.6	1.7	20	42	18	2.44 (0.69–8.61)
** 15–50%**	50.6	8.1	2.4	24	31	21	1.51 (0.82–2.77)
** 50–70%**	18.0	6.4	2.9	34	30	40	0.75 (0.32–1.72)
** >70%**	5.5	6.0	3.3	24	18	56	0.30 (0.05–1.91)

a Scores: Malawi: no facility (0), substandard (1), reduced first level (2), full first level (3), reduced backup (4), full backup (5); Zambia: no facility (0), substandard (1), BEmOC-4 (2), BEmOC-2 (3), BEmOC (4), CEmOC (5).

b test for trend of homogeneity of odds ratios over strata.

We then compared early neonatal mortality between facility births and home births (Table 5, right). In clusters with a low frequency of facility delivery (where thus a large proportion of facility deliveries are complicated cases), there were more early neonatal deaths among babies born in a facility than among babies born at home (OR 1.33 in Malawi, OR 2.44 in Zambia). In clusters with a high frequency of facility delivery (where most births at facilities are normal deliveries), there were less deaths among babies born at a facility than among babies born at home (OR 0.58 in Malawi, OR 0.30 in Zambia). This interaction did not reach significance in either country (p = 0.08 in Zambia), but is also reflected in the fact that overall, facility deliveries (compared to home deliveries) had lower early neonatal mortality in Malawi (OR 0.86) – where half of all births were in a facility – and higher early neonatal mortality in Zambia (OR 1.33) – where only one third of births are in a facility (and thus a higher proportion of facility deliveries are complicated).

## Discussion

We hypothesized that both better geographic accessibility and higher level of delivery care at the closest facility could increase facility use for delivery which would enable prevention and treatment of delivery complications through skilled intrapartum care and thus reduce early neonatal mortality. Higher level of care at the closest facility, in addition to increasing care-seeking, could also reduce early neonatal mortality directly due to a better ability to treat complications, and closer distance could increase care-seeking for post-partum newborn complications and reduce early neonatal mortality that is not intrapartum-related.

However, we found that in Malawi, there was no association between distance to delivery care and early neonatal mortality, and in Zambia, longer distance was associated with higher early neonatal survival. Higher level of care at the closest delivery facility was not associated with early neonatal mortality in either country.

This was despite the fact that longer distance was clearly associated with reduced facility use in both countries. Although distances to the closest delivery care are much shorter in more densely-populated Malawi, the association between delivery care-seeking and distance was even more pronounced than in Zambia, for which we had already shown a distance decay [11]. Higher level of care at the closest delivery facility was not associated with higher facility use in Malawi, unlike in Zambia where we had demonstrated this earlier [11]. This might be because the data for classifying obstetric care in the Malawian Health Facility Census were more limited, resulting in a less reliable assessment of level of care.

To elucidate why longer distance was not associated with higher early neonatal mortality despite being strongly associated with lower facility delivery, we studied the link between facility delivery and early neonatal mortality. A naïve crude comparison showed no significant difference in early neonatal mortality between facility births and home births in both countries, with facility births having slightly higher mortality than home births in Zambia and vice versa in Malawi.

It is clear though, that due to potential confounding by complications during pregnancy or childbirth (Figure 1), the naïve comparison of early neonatal mortality among facility births and home births at the individual level is misleading. Valid data on complications are, however, difficult to get; information collected from women is unfortunately too unreliable to be useful [24], [25].

We thus investigated early neonatal mortality at the cluster level, where we could assume an approximately even distribution of complications. We compared clusters where most women delivered in facilities with clusters where facility delivery was uncommon. The latter clusters were on average farther from a health facility, in line with the results from the individual-level analysis which showed that distance was a strong determinant for facility use. There was, however, no difference in early neonatal mortality between clusters with high and clusters with low levels of facility delivery, thus suggesting that facility delivery may not be effective in decreasing early neonatal mortality.

We could confirm our hypothesis that in settings with low facility use for delivery, women using facilities were more likely to be complicated cases, more frequently seeking hospital care and having caesarean sections (Table 4). This adverse selection of high-risk births into health facilities explains why early neonatal mortality was higher in facilities than at home in these settings – which has been observed in other studies as well [14]. In contrast, in settings where most women delivered in health facilities, early neonatal mortality was lower in health facilities than at home (Table 5). This was not only due to a lower mortality in facilities, having many low-risk normal deliveries as well as complicated cases, but also due to a higher mortality among the few remaining home births, maybe indicating that those left out are at higher risk for other reasons. This pattern was most striking in Zambia, but it is worth noting that none of these trends were statistically significant and that no control for confounding was attempted in this analysis.

The DHS programme has a rigorous process of training and quality assurance, and their data are one of the main sources used to understand health status and care-seeking in low- and middle-income countries [26]–[28]. The JICA HFC has not been carried out in as many settings, yet efforts in Malawi and Zambia did receive considerable technical assistance and it is regarded as “extremely robust” [18]. Nevertheless, this study has certain limitations which may have influenced our findings. The birth histories in the household surveys can contain errors; women may not wish to report sad events and interviewers may fail to record events to avoid asking additional questions. Omission of non-surviving children is most notably of concern for neonatal mortality [29], [30]. Indeed, internal consistency checks of the 2004 Malawi DHS found that births were underreported, especially for non-surviving children, and that in particular, early neonatal mortality appears to be underreported [31]. In the 2007 Zambia DHS, this seemed less of a problem [16]. Early neonatal deaths could furthermore be misreported as stillbirths. Information on stillbirths in the DHS was not sufficiently detailed for using perinatal mortality instead of early neonatal mortality as our main outcome.

The early neonatal mortality risks reported should thus be interpreted with caution. If misreporting was differential, i.e. if women or interviewers at more remote locations were more likely to underreport early neonatal mortality, this could account or partly account for the lack of association between distance and early neonatal mortality, or lead to a reverse association. It could be argued that this is plausible, given that the incentive to reduce one’s workload is bigger for interviewers when their return trip is longer. Furthermore, the likelihood that signs of life in a newborn may go unnoticed and thus lead to misclassification as a stillbirth are probably higher in home births than in facility births, and home births are more common in distant locations, as we have shown. This is in line with the observed attenuation of the reverse distance association in Zambia in the sensitivity analysis using perinatal mortality as the outcome. Finally, only surviving women could be interviewed, which will lead to underestimation of early neonatal mortality where maternal mortality is high.

Further limitations include errors in the distance measurement due to missing or incorrect geographic coordinates, Macro’s geoscrambling procedure [21], missed facilities, the approximation of household coordinates by the cluster centroid and the use of straight-line distance instead of real travel time. All these are likely to be non-differential in regard to early neonatal mortality and would thus underestimate any effects of distance. However, the strong association between distance and facility delivery despite these errors validates the distance measures to some degree. Yet, since we used distance to delivery facilities offering different levels of EmOC, the distance measures are less specific for early neonatal mortality than would be ideal, as other facilities may also provide newborn care for postnatal complications for which we did not have information. Misclassification in level of care, given that we had to make a number of assumptions and given the limited information available especially in Malawi, can have led to underestimation of the effects of level of care on facility delivery and early neonatal mortality. More specific information on quality of care for newborns in particular would have been desirable. Furthermore, the measurement of level of care in the HFC was at one point in time (2002 in Malawi, 2005 in Zambia) during the five-year period with birth data from the DHS (1999–2004 in Malawi, 2002–2007 in Zambia) and it is likely that services will have changed over time. Finally, while we controlled for a wide range of confounders at individual and community level, it is possible that unmeasured factors have caused residual confounding. Lack of public transport, for instance, may compound any harmful effects of distance. It is difficult, however, to conceive of a strong *negative* confounder, i.e. a beneficial factor more common in remote areas, able to disguise an association between longer distance and higher mortality.

Another possible explanation for the lack of association between distance and early neonatal mortality is that the chain of events leading from one to the other is long and influences are acting on an increasingly smaller percentage of births. While there can also be a direct effect of distance on early neonatal mortality through care-seeking for sick newborns, facility delivery is seen as the main intermediate factor (Figure 1). In Malawi, where the association between distance and facility delivery is strongest, the proportion of facility deliveries in locations farthest from a facility (>15 km) is 28% and closest (<2 km) it is 68% (Table 2B). This difference of 40% is the maximum number of births on which any expected beneficial effect of facility delivery can act to produce an effect of distance on mortality. In Zambia, this number is smaller, less than 20%. The vast majority of these babies will be fine, only some will develop complications. Some complications are not amenable to intrapartum and postnatal care available in low-income settings anyway, e.g. congenital anomalies or complications due to very preterm birth. Even if there is a beneficial effect of facility delivery on early neonatal mortality due to prevention and treatment of intrapartum complications and of infections, this effect on a small percentage of babies may get diluted in the larger numbers and we thus may not observe any association between distance and mortality.

It has been estimated that in high and very high mortality settings, about 11% of early neonatal deaths could be prevented if all births currently in facilities had access to comprehensive emergency obstetric care and newborn resuscitation (i.e. filling the “quality gap”), and about 23% could be saved if 90% of births had access to such care (i.e. filling the “quality gap” and the “coverage gap”), assuming that these interventions act mainly on the one third which are intrapartum-related causes of death [32]. This highlights that the relatively smaller difference in coverage of facility delivery between distant and close locations in our settings cannot be expected to reduce early neonatal mortality by a large amount, even more so since most facilities offer only very limited emergency obstetric or newborn capabilities (Table 1 and [19]).

A case-control study in northern Vietnam [9], to our knowledge the only other study on the effect of distance to health facilities on neonatal mortality, found a strong association between mortality and longer distance, although distances were very short in that setting: the closest health facility was on average 1 km away, and the closest district hospital 7 km. Around 80% of births were in health facilities; interestingly, the association between distance and mortality persisted after adjusting for place of delivery [9]. The authors speculate that facilities in more remote areas offer a lower quality of care which may contribute to the observed distance effect [9]. In this setting, where most deliveries are in facilities, neonatal mortality was lower among facility births than among home births [12].

In contrast, a study in Indonesia, where most women deliver at home, found that early neonatal mortality was higher among facility births than unattended home births, particularly in rural areas, which the authors attributed to poor access to care and low quality of health services [13]. While they controlled for *reported* delivery complications, it is likely that there was residual confounding by complications and the results are thus difficult to interpret. A study from Tanzania found no difference in neonatal mortality between home and facility births and also concluded this was due to low quality of care at facilities, dismissing the possibility of confounding by complications [33]. The study from Bangladesh mentioned earlier [14] had data over a period of 19 years during which facility delivery became more common. Maternal mortality, stillbirths and early neonatal mortality were higher in facilities than at home throughout, but decreased over time – consistent with self-selection of complicated births into facilities and increasing dilution by more uncomplicated births in facilities. To circumvent the problem of confounding by complications, in the future, studies comparing mortality between facility and home births may want to consider studying the association also on the cluster (or community) level, in addition to the individual level.

In order to achieve a reduction in early neonatal deaths, as well as in stillbirths and maternal deaths, it is clear that provision of “effective maternal and neonatal health services” is needed to overcome current gaps in care at birth [5]. Besides deficiencies in quality of care for mothers, health facilities may have even larger deficits in the quality of care provided to newborns – which could be a reason for the lack of association between cluster-level facility delivery and early neonatal mortality found in this study (Table 5). The overall very low proportion of caesarean sections in our samples from rural Zambia and rural Malawi, 1.5% and 2.5% respectively, are not even sufficient to cover maternal indications, and C-sections are thus probably rarely done for fetal indications. If the focus even in referral-level facilities is mainly on saving the mother’s life, not the newborn’s, this could also explain the lack of association between level of care at the closest facility and early neonatal mortality. Finally, it is also possible that poor infection control in health facilities or lack of support for breastfeeding or thermal control may even increase mortality.

### Conclusions

Although proximity to delivery care was strongly associated with higher facility use for delivery, it was not associated with lower early neonatal mortality, suggesting that facility use may not necessarily translate into mortality reduction. We show that available data can be used for such analyses, however, the reliability of these data can be questioned, in particular the reporting of early neonatal deaths in the DHS. Studies using alternative data, e.g. from demographic surveillance sites, are indicated.

Nevertheless, it would be helpful if routinely collected national datasets such as the DHS and health facility censuses could be used to monitor improvements in maternal and newborn care. Measuring outcome indicators, such as presence of a health professional at delivery, is not sufficient, we also need information upstream on health system output indicators (e.g. coverage with obstetric and newborn services) to know where the problem lies and thus where improvements are needed [34], [35] and eventually downstream on health impact indicators to monitor whether better indicators upstream indeed translate into reduced mortality.

To achieve this, a number of improvements in these data are required. Inclusion of more details on stillbirth histories in the DHS would enable calculation of perinatal mortality and circumvent some problems with misclassification of early neonatal deaths. Alternatives to the scrambling of geodata should be identified to enable more precise distance measurements. And finally, it would be helpful if a set of signal functions for obstetric and newborn care could be agreed upon and be collected in future health facility assessments to enable more precise classification of facility functioning and to some degree quality of care, as recently suggested [36].

## Supporting Information

Table S1 **List of confounders considered in the statistical analysis of the associations between distance to care and i) early neonatal mortality, ii) facility delivery.**
(DOC)

## References

[1] Lawn JE, Cousens S, Zupan J (2005) 4 million neonatal deaths: when? Where? Why? Lancet 365: 891–900. 15752534 10.1016/S0140-6736(05)71048-5

[2] Black RE, Cousens S, Johnson HL, Lawn JE, Rudan I, et al (2010) Global, regional, and national causes of child mortality in 2008: a systematic analysis. Lancet 375: 1969–1987. 20466419 10.1016/S0140-6736(10)60549-1

[3] UNICEF, WHO, The World Bank, United Nations Population Division (2012) Levels and Trends in Child Mortality. New York. Available: http://www.childmortality.org/.

[4] Lozano R, Wang H, Foreman KJ, Rajaratnam JK, Naghavi M, et al (2011) Progress towards Millennium Development Goals 4 and 5 on maternal and child mortality: an updated systematic analysis. Lancet 378: 1139–1165. 21937100 10.1016/S0140-6736(11)61337-8

[5] Lawn JE, Lee AC, Kinney M, Sibley L, Carlo WA, et al.. (2009) Two million intrapartum-related stillbirths and neonatal deaths: where, why, and what can be done? Int J Gynaecol Obstet 107 Suppl 1: S5–18, S19. 10.1016/j.ijgo.2009.07.016 19815202

[6] Okwaraji YB, Cousens S, Berhane Y, Mulholland K, Edmond K (2012) Effect of geographical access to health facilities on child mortality in rural ethiopia: a community based cross sectional study. PLoS One 7: e33564. 22428070 10.1371/journal.pone.0033564 PMC3299799

[7] Rutherford ME, Dockerty JD, Jasseh M, Howie SR, Herbison P, et al (2009) Access to health care and mortality of children under 5 years of age in the Gambia: a case-control study. Bull World Health Organ 87: 216–224. 19377718 10.2471/BLT.08.052175 PMC2654650

[8] Schoeps A, Gabrysch S, Niamba L, Sie A, Becher H (2011) The effect of distance to health-care facilities on childhood mortality in rural Burkina Faso. Am J Epidemiol 173: 492–498. 21262911 10.1093/aje/kwq386

[9] Malqvist M, Sohel N, Do TT, Eriksson L, Persson LA (2010) Distance decay in delivery care utilisation associated with neonatal mortality. A case referent study in northern Vietnam. BMC Public Health 10: 762. 21144058 10.1186/1471-2458-10-762 PMC3009650

[10] Lawn J, Shibuya K, Stein C (2005) No cry at birth: global estimates of intrapartum stillbirths and intrapartum-related neonatal deaths. Bull World Health Organ 83: 409–417. 15976891 PMC2626256

[11] Gabrysch S, Cousens S, Cox J, Campbell OM (2011) The influence of distance and level of care on delivery place in rural zambia: a study of linked national data in a geographic information system. PLoS Med 8: e1000394. 21283606 10.1371/journal.pmed.1000394 PMC3026699

[12] Malqvist M, Nga NT, Eriksson L, Wallin L, Ewald U, et al (2008) Delivery care utilisation and care-seeking in the neonatal period: a population-based study in Vietnam. Ann Trop Paediatr 28: 191–198. 18727847 10.1179/146532808X335633

[13] Titaley CR, Dibley MJ, Roberts CL (2011) Type of delivery attendant, place of delivery and risk of early neonatal mortality: analyses of the 1994–2007 Indonesia Demographic and Health Surveys. Health Policy Plan. 10.1093/heapol/czr053 21810892

[14] Ronsmans C, Chowdhury ME, Koblinsky M, Ahmed A (2010) Care seeking at time of childbirth, and maternal and perinatal mortality in Matlab, Bangladesh. Bull World Health Organ 88: 289–296. 20431793 10.2471/BLT.09.069385 PMC2855602

[15] Birthplace in England Collaborative Group (2011) Perinatal and maternal outcomes by planned place of birth for healthy women with low risk pregnancies: the Birthplace in England national prospective cohort study. BMJ 343: d7400. 22117057 10.1136/bmj.d7400 PMC3223531

[16] Central Statistical Office, Ministry of Health, Tropical Diseases Research Centre, University of Zambia, Macro International Inc. (2009) Zambia Demographic and Health Survey 2007. Calverton, Maryland, USA: CSO and Macro International Inc. Available: http://www.measuredhs.com/publications/publication-FR211-DHS-Final-Reports.cfm.

[17] Rutstein SO, Rojas G (2006) Guide to DHS statistics. Calverton, Maryland: ORC Macro, Demographic and Health Surveys. Available: http://www.measuredhs.com/pubs/pdf/DHSG1/Guide_DHS_Statistics.pdf.

[18] Health Facility Assessment Technical Working Group (2005) Profiles of Health Facility Assessment Methods. MEASURE Evaluation, USAID. Available: http://www.cpc.unc.edu/measure/tools/monitoring-evaluation-systems/hfa-methods/.

[19] Gabrysch S, Simushi V, Campbell OM (2011) Availability and distribution of, and geographic access to emergency obstetric care in Zambia. Int J Gynaecol Obstet 114: 174–179. 21669427 10.1016/j.ijgo.2011.05.007

[20] WHO (2010) Screening Donated Blood for Transfusion-Transmissible Infections. Recommendations. Geneva. Available: http://www.who.int/bloodsafety/ScreeningDonatedBloodforTransfusion.pdf. 23741773

[21] MEASURE DHS What We Do. Geographic Information Systems (GIS). Available: http://www.measuredhs.com/What-We-Do/GIS.cfm. Accessed: 30 March 2012.

[22] Adisasmita A, Deviany PE, Nandiaty F, Stanton C, Ronsmans C (2008) Obstetric near miss and deaths in public and private hospitals in Indonesia. BMC Pregnancy Childbirth 8: 10. 18366625 10.1186/1471-2393-8-10 PMC2311270

[23] Filippi V, Ronsmans C, Gohou V, Goufodji S, Lardi M, et al (2005) Maternity wards or emergency obstetric rooms? Incidence of near-miss events in African hospitals. Acta Obstet Gynecol Scand 84: 11–16. 15603561 10.1111/j.0001-6349.2005.00636.x

[24] Bell J, Curtis SL, Alayón S (2003) Trends in delivery care in six countries. Calverton, Maryland USA. Available: http://www.measuredhs.com/publications/publication-AS7-Analytical-Studies.cfm.

[25] Glei DA, Goldman N, Rodriguez G (2003) Utilization of care during pregnancy in rural Guatemala: does obstetrical need matter? Soc Sci Med 57: 2447–2463. 14572850 10.1016/s0277-9536(03)00140-0

[26] Johnson K, Grant M, Khan S, Moore Z, Armstrong A, et al. (2009) Fieldwork-Related Factors and Data Quality in the Demographic and Health Surveys Program. Calverton, Maryland: ICF Macro. Available: http://measuredhs.com/publications/publication-AS19-Analytical-Studies.cfm.

[27] MEASURE DHS Demographic and Health Surveys. Available: http://www.measuredhs.com/aboutsurveys/dhs/start.cfm. Accessed 2012 Oct 14.

[28] Pullum TW (2008) An Assessment of the Quality of Data on Health and Nutrition in the DHS Surveys, 1993–2003. Calverton, Maryland. Available: http://measuredhs.com/publications/publication-MR6-Methodological-Reports.cfm.

[29] Curtis S (1995) Assessment of the quality of data used for direct estimation of infant and child mortality in DHS-II surveys. Occasional Papers No.3. Calverton, Maryland: Macro International Inc. Available: http://measuredhs.com/publications/publication-op3-occasional-papers.cfm.

[30] Neal S (2012) The measurement of neonatal mortality: How reliable is Demographic and Household Survey Data? Working Paper 25. ESRC Centre for Population Change Available: http://www.cpc.ac.uk/publications/home.php.

[31] National Statistical Office (NSO) [Malawi], ORC Macro (2005) Malawi Demographic and Health Survey 2004. Calverton, Maryland: NSO and ORC Macro. Available: http://www.measuredhs.com/publications/publication-FR175-DHS-Final-Reports.cfm.

[32] Lawn JE, Kinney M, Lee AC, Chopra M, Donnay F, et al (2009) Reducing intrapartum-related deaths and disability: can the health system deliver? Int J Gynaecol Obstet 107 Suppl 1S123–142. 19815205 10.1016/j.ijgo.2009.07.021

[33] Nathan RD, Mwanyangala MAM (2012) Survival of neonates in rural Southern Tanzania: does place of delivery or continuum of care matter? BMC Pregnancy Childbirth 12: 18. 22439592 10.1186/1471-2393-12-18 PMC3384458

[34] Gabrysch S, Zanger P, Campbell OM (2011) Emergency obstetric care availability: a critical assessment of the current indicator. Trop Med Int Health 17: 2–8. 21831117 10.1111/j.1365-3156.2011.02851.x

[35] Gabrysch S, Zanger P, Seneviratne HR, Mbewe R, Campbell OMR (2011) Tracking progress towards safe motherhood: meeting the benchmark yet missing the goal? An appeal for better use of health-system output indicators with evidence from Zambia and Sri Lanka. Tropical Medicine and International Health 16: 627–639. 21320245 10.1111/j.1365-3156.2011.02741.x

[36] Gabrysch S, Civitelli G, Edmond KM, Mathai M, Ali M, et al.. (2012) New signal functions to measure the ability of health facilities to provide routine and emergency newborn care. PLoS Med (in print). 10.1371/journal.pmed.1001340 PMC3496666 23152724

