# Supplementary material for: Distance to Care, Facility Delivery and Early Neonatal Mortality in Malawi and Zambia
Source: PLoS One. 2012 Dec 27;7(12):e52110. doi: 10.1371/journal.pone.0052110 (PMC3531405; doi:10.1371/journal.pone.0052110)
Supplement: Table S1 — List of confounders considered in the statistical analysis of the associations between distance to care and i) early neonatal mortality, ii) facility delivery. (DOC) [file pone.0052110.s001.doc]

**Supporting information**

**Table S1.** List of confounders considered in the statistical analysis of the associations between distance to care and i) early neonatal mortality, ii) facility delivery.

| **Potential confounders** | **Distance and early neonatal mortality** | **Distance and facility delivery** |
| --- | --- | --- |
| **Baby level** |  |  |
| Birth order | X | X |
| Twin | X | X |
| Newborn size estimate | X |  |
| Sex | X |  |
|  |  |  |
| **Birth level** |  |  |
| Number of siblings below 7 years of age | X | X |
| Wantedness of pregnancy | X | X |
|  |  |  |
| **Mother level** |  |  |
| Age | X | X |
| Literacy | X | X |
| Education | X | X |
| Occupation | X | X |
| Language | X | X |
| Ethnic group | X | X |
| Religion | X | X |
| Marital status | X | X |
| Husband’s education | X | X |
| Husband’s occupation | X | X |
| Relationship autonomy | X | X |
| Exposure to health information in the media | X | X |
| Frequency of media use | X | X |
| Fertility attitudes | X | X |
|  |  |  |
| **Household level** |  |  |
| Wealth (asset index) | X | X |
|  |  |  |
| **Cluster level** |  |  |
| Men’s opinion on women’s autonomy | X | X |
| Men’s media use | X | X |
| Men’s fertility attitudes | X | X |
| Women’s financial autonomy | X | X |
| Women’s health-care seeking autonomy | X | X |
| Women’s mobility autonomy | X | X |
| Women’s relationship autonomy | X | X |
| Women’s media use | X | X |
| Women’s fertility attitudes | X | X |
